# An interventional study for improving the manual dexterity of dentistry students

**DOI:** 10.1371/journal.pone.0211639

**Published:** 2019-02-01

**Authors:** Diva Lugassy, Yafi Levanon, Nir Shpack, Shifra Levartovsky, Raphael Pilo, Tamar Brosh

**Affiliations:** 1 Department of Oral Biology, The Maurice and Gabriela Goldschleger School of Dental Medicine, Sackler Faculty of Medicine, Tel Aviv University, Tel Aviv, Israel; 2 Department of Occupational Therapy, The Stanley Steyer School of Health Professions, Sackler Faculty of Medicine, Tel Aviv University, Tel Aviv, Israel; 3 Department of Orthodontics, The Maurice and Gabriela Goldschleger School of Dental Medicine, Sackler Faculty of Medicine, Tel Aviv University, Tel Aviv, Israel; 4 Department of Oral Rehabilitation, The Maurice and Gabriela Goldschleger School of Dental Medicine, Sackler Faculty of Medicine, Tel Aviv University, Tel Aviv, Israel; Meridional Faculty IMED, BRAZIL

## Abstract

**Objectives:**

Traditionally, the acquisition of manual skills in most dental schools worldwide is based on exercises on plastic teeth placed in a "phantom head simulator". No manual trainings are done at home. Studies revealed that preliminary training of one motoric task leads to significant improvement in performance of the required motoric task that has similar components. Performing tasks indirectly via a dental mirror are complicated for the young dental students. We hypothesized that instructed training of basic skills required in dentistry at home on a tool simulating the phantom laboratory will improve the capabilities of the students and will be reflected by their clinical grades.

**Methods:**

We developed a portable tool PhantHome which is composed of jaws, gingival tissue, rubber cover and a compatible stand. Specific teeth produced by a 3D printer with drills in different directions were placed in both jaws. Students were requested to insert pins by using tweezers and dental mirror according to instructions initiating with easy tasks and continue to ones that are more complicated. 106 first clinical year dental students participated in the study; 65 trained only in the traditional phantom lab (control). 41 trained at home by the PhantHome tool two weeks before and 2 months during the initial stage of phantom lab. The students grades routinely provided in the phantom laboratory at different stages were compared.

**Results:**

Students who trained with the portable tool performed better than the control group in the first direct and second indirect preparations (p<0.05). These exams were taken when the PhantHome was available to the students. Then, the tool was returned and the phantom course continued regularly. We believe that this is why no differences between the grades of the groups were observed further on.

**Conclusions:**

Training by the PhantHome improves motor skills and consequently the clinical performances.

## 1. Introduction

Most medical professions require manual capabilities, in particular surgeons, including dental surgeons that have to demonstrate a high ability of manual dexterity skills and high level of proprioception recognition in every treatment. Therefore, dental students have to acquire comprehensive theoretical knowledge as well as specific manual skills during their early dental education. This capability is developed by training on artificial plastic teeth placed in a "phantom head simulator"[1]. This training includes clinical tasks using direct vision and indirect vision via a dental mouth mirror.

Although dexterity can be improved after training over the course of time [2] ("practice makes perfect"[3]), a considerable number of students are not allowed to advance to the clinical studies and treat patients due to inadequate performance in pre-clinical simulation courses [4]. Accordingly and as manual performance can be improved by means of a positive transfer of motor learning, training tools to enhance performance in preclinical courses have been developed [5–7].

Performing tasks using indirect vision is significantly more difficult to perform and acquire (~50%) as compared to tasks performed using direct vision [8]. Thus, researchers developed various methodologies for practicing that skill [5–7]. For example, an indirect training device, named Mirroprep, consists of a high-profile quality steel sheet with a mirror mounted to its rearmost wall and a replica of a dental drill [6]. The subject follows curved tracks on a pad bounded by two lines with the pencil. The results showed that mirror vision can be improved and a high degree of transfer to other manual exercises can be obtained. Another methodology consisted of 12 diagrams that had been printed on heavy weight paper and fitted to the upper arch of the dentoform. A pencil was inserted in the head of the low speed turbine and the students were instructed to trace the various diagrams using mouth mirror [5]. It was concluded that repeated practice with progressively ascending level of difficulty in tracing has a great effect on the development of indirect vision skills for performing cavity preparation.

Recent technological advances have created a diverse range of virtual reality simulators that can facilitate learning and evaluation in numerous areas of medical education [9–11]. Some dental schools utilize virtual reality simulators for training, especially during the pre-clinical years of studies [12–18]. Research has confirmed the effectiveness of such simulators over the traditional phantom lab [13,17], primarily in terms of self-learning manual skills, which lead to reduction in manpower tutoring. However, most studies have claimed that this innovation should not be used alone, and should be supplemented by the traditional phantom laboratory [19–21]. Unfortunately, the high costs of the advanced technologies [12] prevent implementation in most of the dental schools in the world.

In the current study, it was hypothesized that practice at home on a portable tool that has general principles and similar elements as the phantom laboratory should lead to significantly improved performance in the phantom laboratory (transfer of learning). The skills that are incorporated in this training are hand-eye coordination, fine motor skill, spatial perception and accuracy. Therefore, the aims of the current study were:

1. To develop a portable kit with compatible training program for home training.
2. To assess whether training using the portable tool in parallel to the phantom course will improve students’ performance (transfer test).

## 2. Methods

### 2.1 Study tools

#### PhantHome—The portable training tool (Fig 1)

**Fig 1:**
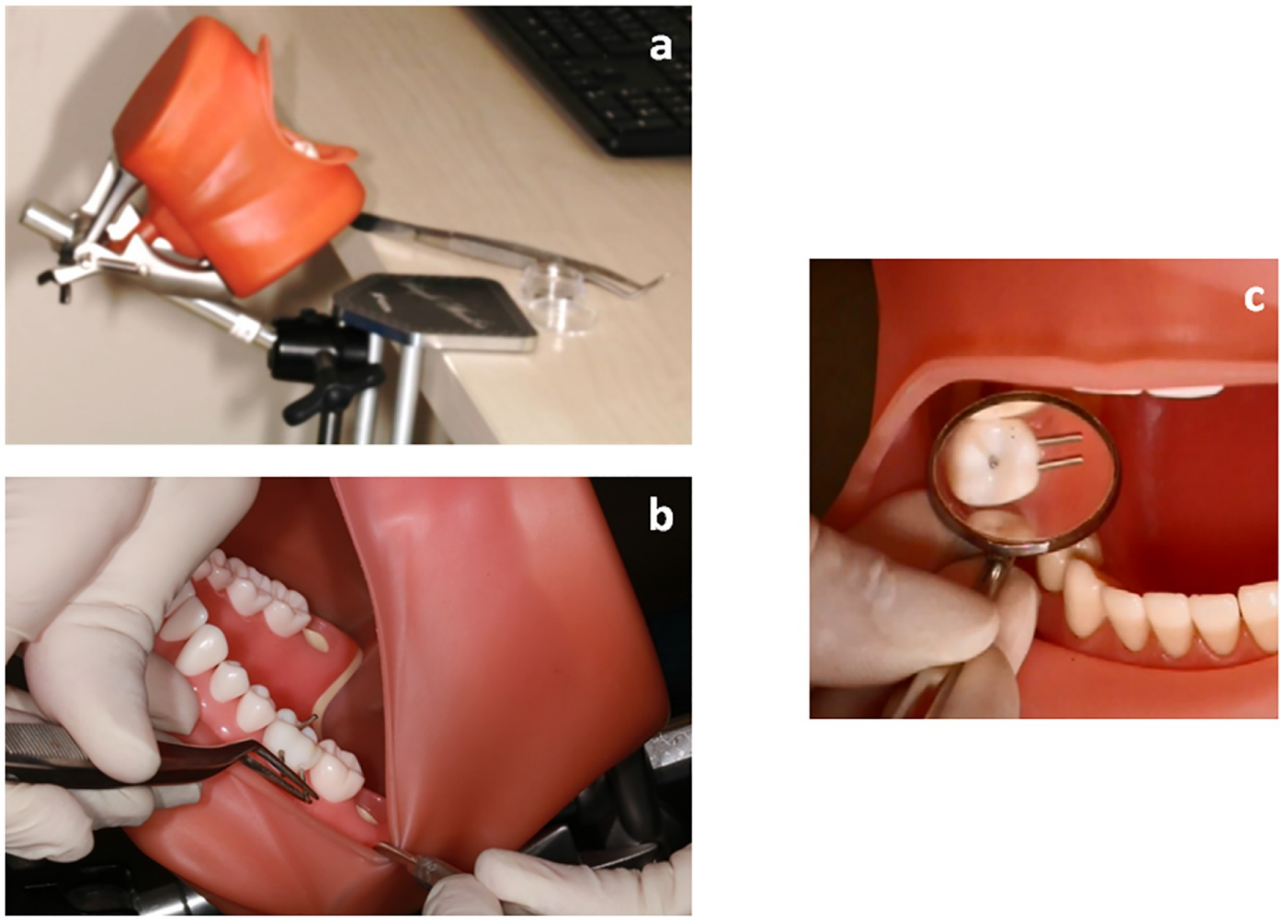
The PhantHome—Novel portable tool. (a) The tool is attached to a desk. (b) (c) "Intra-oral" view of the teeth with inserted pins.

The principles of the current tool are based on manual dexterity test from occupational therapy called O’Connor Tweezer Dexterity Test. This test [8,22,23] consists of a board having 100 holes and a cup that holds 100 pins. The participant inserts all 100 pins into the holes using tweezers with the dominant hand. The score is the time needed to do the mission. The O’Connor test was modified also to be used with indirect vision and was previously reported to be appropriate for assessing manual skills of dentistry students [8]. The current portable interventional tool is composed of a commercial phantom head containing of two jaws with plastic teeth, artificial gingival tissue and a rubber cover, which simulates the mouth of the patient (Nissin Dental Products INC, Nakagoku, Japan). Specific teeth in both jaws were replaced by preplanned teeth manufactured by 3D printing technology utilizing Polyjet^™^ jetting technology (Objet 3D printer, Stratasys Ltd, Minesota US), in which drills in different directions were incorporated. The students were instructed to insert matching metal pins (1mm diameter, 10mm length) in a specific order into these prepared pin holes. The portable tool was accompanied by training instructions. The tasks were divided into several steps, in ascending order of difficulty. Thus the students inserted pins initially in the teeth of the lower arch by direct vision and gradually increasing the level of difficulty by inserting pins in the teeth of the upper arch by using a dental mirror. The students were instructed to practice at least twice a week and to report the amount of training they did. They were also advised to continue practicing by inserting the pins alternately to both arches. (S1 Appendix). The PhantHome tool can be attached to any table with a compatible stand. All the components of the kit are organized in a small carrying box.

### 2.2 The study groups

106 first clinical year dental students from the phantom head lab course at Tel Aviv University, Israel, participated in the study. Experimental group: 41 students as experimental group from the 2017 academic year (26 females, 15 males, age 25.51±2.24 years, range 23–40 years) and 65 as historical control group from the 2015 and 2016 academic years (42 females, 23 males, age 25.45±2.79 years, range 23–40 years). Six students from the control group dropped-out at the early stage of the phantom course and were excluded from the analysis. Participants’ inclusion criteria were students of the 1^st^ clinical year. Exclusion criteria: previous exposure to training with a phantom head simulator. Each participant was requested to complete a questionnaire regarding age, gender, hours of sleep at night and the dominant hand. Governmental psychometric score defined the cognitive ability.

### 2.3 Study procedure

The Ethics Committee of Tel Aviv University specifically approved this study. All participants signed informed consent. Both groups were assessed prior commencing the phantom course for their basic fine motor skills and spatial perceptions using the O’Connor Tweezer Dexterity Test in direct vision (O-D) and in indirect vision (O-IND), respectively. These tests were conducted at two time-points: at the beginning (T0) and at the end of the 1^st^ clinical year (T1).

The control group (N = 59) underwent phantom practice without access to the PhantHome training tool. The experimental group received the PhantHome training tool to practice at home two weeks before the beginning of the academic year until the end of the first two months of the phantom course. Those students were highly motivated and goal oriented for using the novel portable tool. The phantom course continued regularly as with the previous years. These students constituted the entire student group of that year, thus avoiding selection bias. Due to the limitations inherent in a before-and-after study, demographic characteristics, cognitive, basic fine motor skills and spatial ability were compared between the experimental and control groups.

#### Clinical performances

**Fig 2:**
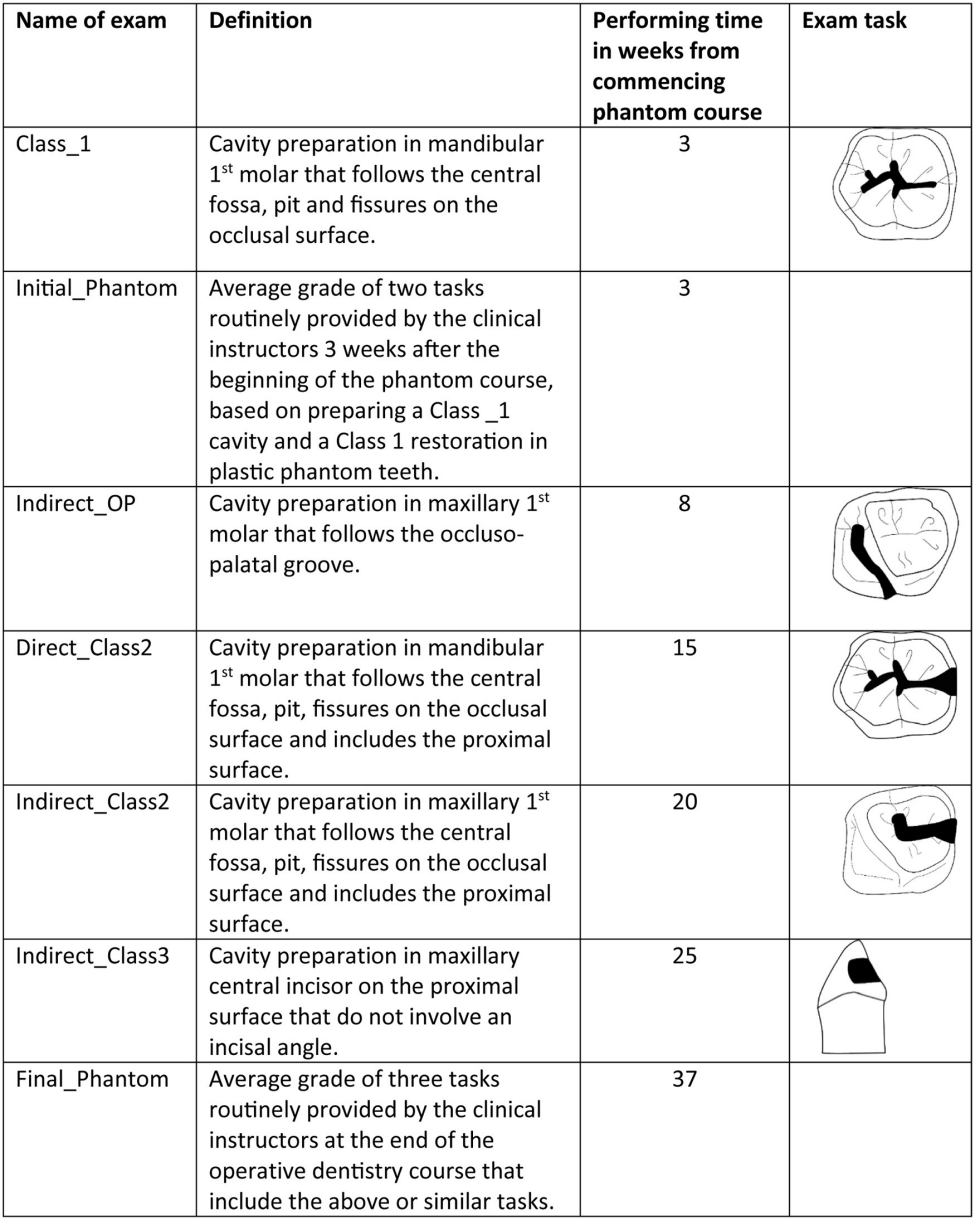
Time table and illustration of the exams’ tasks along the phantom course.

The students’ grades routinely provided in the phantom laboratory for different preparation tasks (i.e., drilling using dental turbine in plastic teeth) were compared between the control and experimental groups for various preparations, starting with simple tasks at the beginning of the course, followed by more complicated ones during the year (Fig 2). Three instructors graded each test using several common criteria: 1. The outer outline, the dimension and depth of the cavity preparation compared to the pre-determined requirements according to the principals of minimal cavity preparation. 2. Orientation of the cavity relative to the longitudinal axis of the tooth, and 3. Finishing of the cavity walls. Each of the above criteria has predetermined specific score weight known to the instructors and the students. The mean of the three instructors’ scores is considered. The grades are on a continuous scale of 0–100 and the passing grade is 60. Since student identification numbers were coded, the instructors were blinded to their identity. The principal investigator who conducted the O’Connor test at the beginning of the study was blind to the students’ clinical grades for the phantom course. In addition, 2 other parameters were compared between the groups: the initial phantom grade (Initial.Phantom) and the final phantom grade (Final.Phantom). Initial.Phantom is the average grade routinely provided by the clinical instructors three weeks after the beginning of the phantom course, and is based on preparing a Class_1 cavity and an occlusal restoration in plastic phantom teeth. Final.Phantom is the average grade of the three tasks (preparations and restorations) routinely provided by the clinical instructors at the end of the operative dentistry course (Fig 2).

### 2.4 Statistics

Descriptive statistics of both groups was obtained regarding independent variables of age, gender, basic fine motor skills, spatial perception, cognitive ability, dominant hand and hours of sleep at night. Independent t-test and chi-square tests compared the control and experimental groups for compatibility in those variables. The manual grades of the groups were compared by an independent-samples t-test after performing the Shapiro-Wilk test, which showed a normal distribution of the grades. Comparisons between the O-D and O-IND were performed using paired t tests at different times.

Significant differences were considered as p<0.05.

## 3. Results

### 3.1 Comparing characteristics of the control and experimental groups

No statistically significant differences were found between the experimental and control groups with regard to age, gender, basic fine motor skills, spatial perception, cognitive ability, hours of sleep at night and the dominant hand (p>0.05), (S2 Appendix).

### 3.2 O’Connor Tweezer Dexterity Test

**Fig 3:**
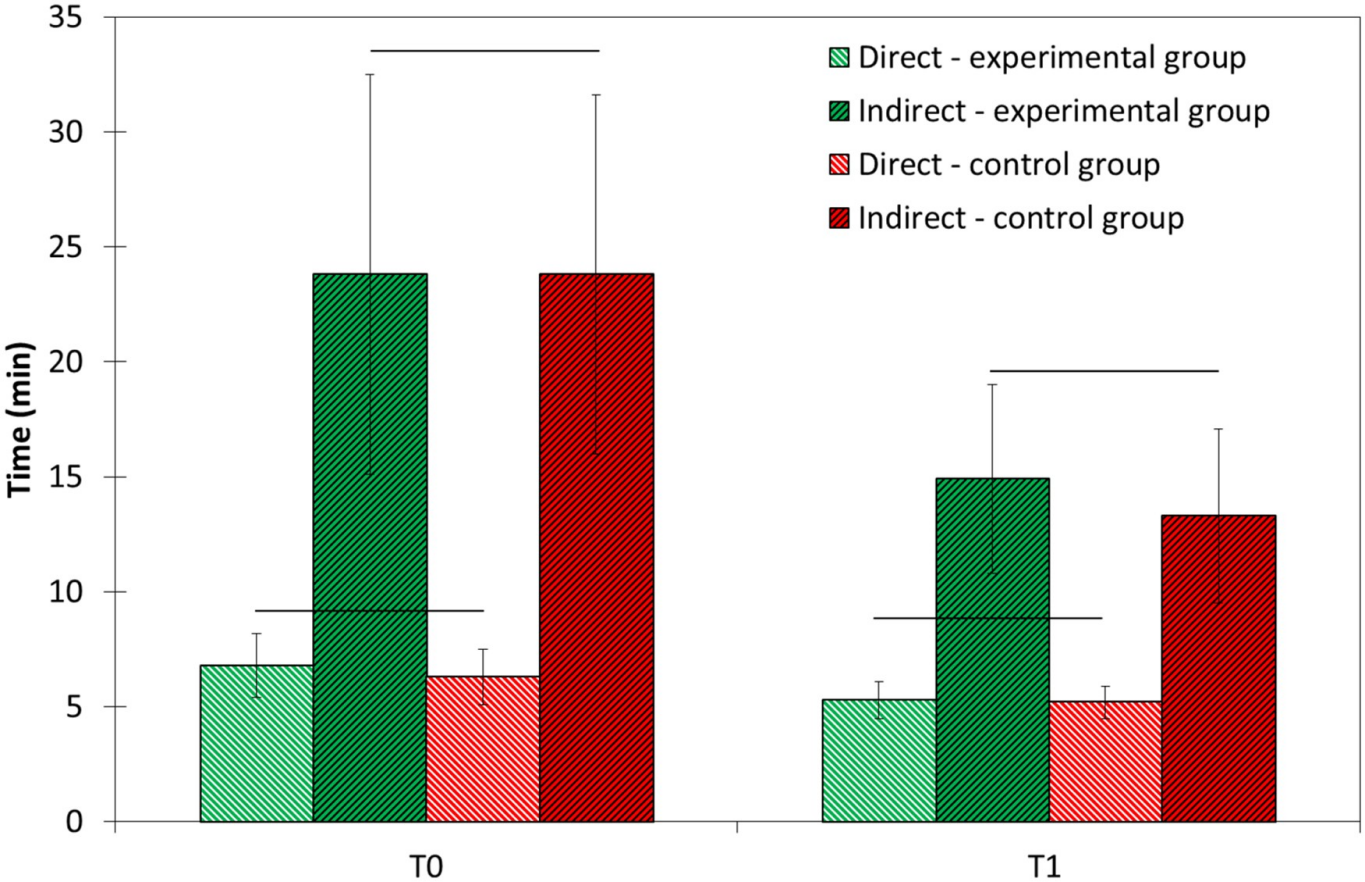
Mean scores of O’Connor Tweezer Dexterity Test at the beginning (T0) and at the end (T1) of the 1th clinical year (N = 41) and control (N = 59) groups in direct and indirect vision. Horizontal lines between the experimental and control groups at T0 and T1 for O’Connor direct and O’Connor indirect represent no significant differences between the groups. Significant differences were found between direct and indirect scores (p<0.0005) at each tested time for each group. Significant differences were found between T0 and T1 for the same exam for each group (p<0.0005).

No statistical differences were found between the groups at T0 and at T1 in the scores of the O-D and O-IND tests (p>0.05). In both groups, performance times under O-D were significantly shorter as compared to O-IND (p<0.0005) at both time points. Also, in both groups the scores at T1 significantly decreased in direct and indirect vision compared to T0 (p = 0.0005): by ~20% for O-D and by 40% for O-IND (Fig 3).

### 3.3 Clinical grades in the phantom course

**Fig 4:**
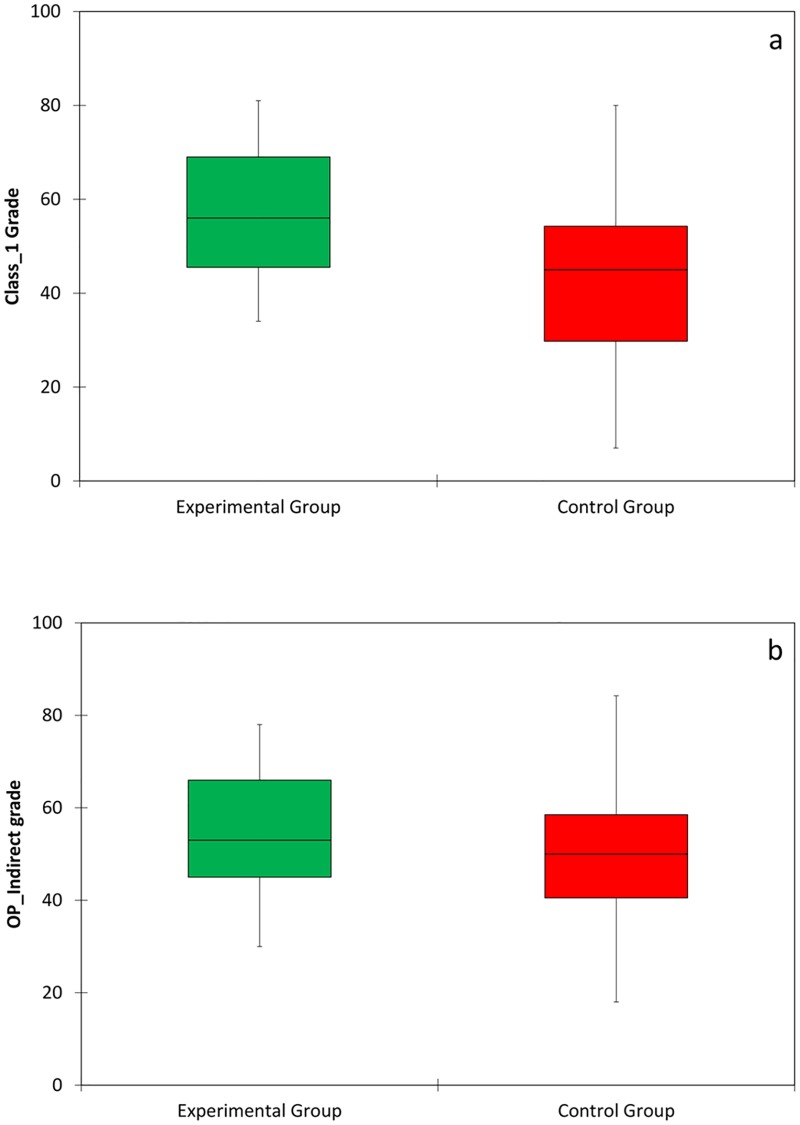
Box diagrams of the (a) Class_1 and (b) OP_indirect grades of the experimental (N = 41) and control (N = 59) groups. Horizontal bar within each box represents the median.

**Fig 5:**
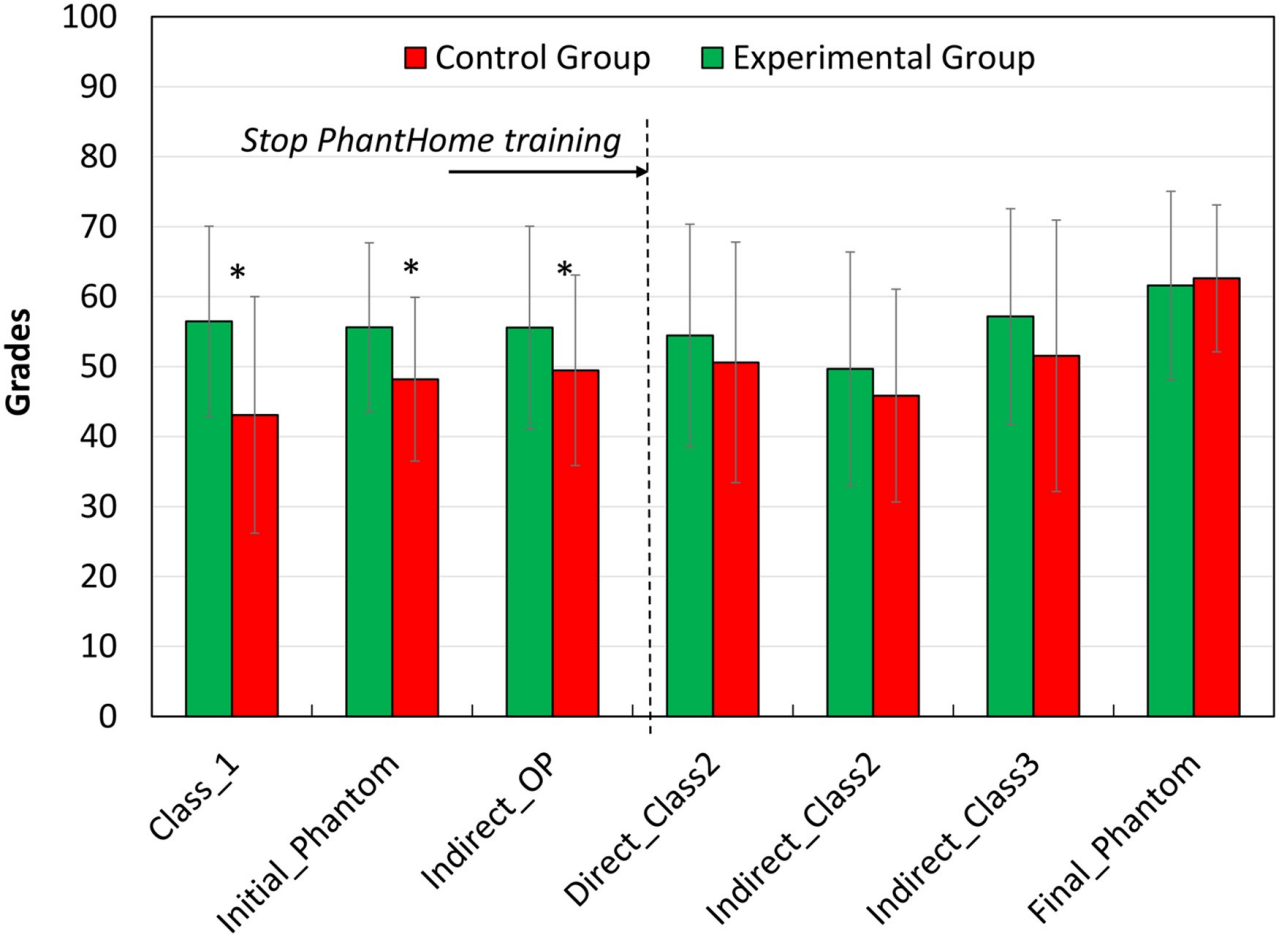
Mean clinical grades of phantom exams of the experimental (N = 41) and control (N = 59) groups during the phantom course. * Significant differences between mean values of the groups for the specific exam (p<0.05).

Fig 4(a) and 4(b) presents the conditional probability distribution of the Class_1 and OP_indirect scores exams. Significant differences were found between the experimental and control groups in Class_1 (direct exercise, p = 0.0005), Indirect_OP (p = 0.045) preparations and Initial.Phantom (p = 0.004). In all these exercises the experimental group performed better. These tests were performed while the PhantHome tool was available only to the experimental group at home. However, there were no significant differences between the groups’ grades in the following exams of the course, carried out after the experimental group stopped the extra training with the PhantHome tool (Fig 5).

## 4. Discussion

In this study, a novel portable tool, named "PhantHome" was developed for the purpose of home training for dentistry students in basic skills required in the phantom course. Training with a tool that simulates a patient’s mouth is based on the transfer of learning theory, according to which repeated training of one motoric task leads to significant improvement in performance of another motoric task that has similar components [24,25]. It has long been recognized that the extent of transfer of training between one learning task to another depends on the similarity of the tasks [26]. Therefore, to assure a high degree of transfer from a motoric activity learned in the PhantHome tool to motoric tasks required in the phantom course, both tasks must have comparable components. Although this home training, that includes the insertion of small pins by using tweezers and dental mirrors on similar artificial jaws, doesn’t simulate cavity preparation, it trains some of the components needed in cavity preparation such as orientation in the oral cavity, manipulation of instruments in direct vision and especially in indirect vision and grip control. The results of our study prove our hypothesis that practice at home on a portable tool that has general principles and similar elements as the phantom laboratory indeed lead to improve performances in the phantom laboratory (transfer of learning).

The results of our study show that adding home training with the novel tool two weeks before and two months during the initial phantom studies led to significantly higher clinical grades as compared to the students who didn’t trained at home. However, the benefit achieved was detected only in the early stages of the phantom course, whereas in the advanced stages there were no significant differences between the experimental and control groups (Fig 5). This finding is consistent with the results of similar studies that examined the impact of preliminary training with a simpler task whose components are similar to those of the required task. These studies showed significant improvement in the experimental group in the early stages of training, as a result of the initial preliminary training. However, after repeated training, the gap between the experimental and control groups gradually decreased and disappeared [27–29]. The purpose of those studies was not to improve the final result of the required task, but rather to examine the extent of transfer [30], which lead to reduced time needed to reach a similar final result. For example, Gagne et al. [28] concerning the field of occupational therapy investigated the extent of transfer in learning of a complex motor skill, when varying amounts of initial practice are given for a task that is itself a component activity of the required skill. Their research provides evidence of a relationship between the amount of preliminary practice and the learning of a complex motor skill. This transfer is effective especially during the initial stages of learning; while at the end of the training, the differences between the groups are reduced and negligible. The authors concluded that the extent of learning transferred and the ease of learning a complex skill depend both on the degree of similarity between the tasks and on the amount of previous practice. The transfer phenomena was also observed in the results of the O’Connor Tweezer Test. Both direct and indirect missions were improved after the year of intensive manual clinical tasks, however, the influence was twice pronounced in the indirect mission. This is reasonable as ordinary people don’t need to perform tasks in indirect view and thus their capabilities are low as found in T0. But, after training in the phantom course where exercises have to be done via dental mirrors in indirect view, the performances were significantly improved (Fig 3). Thus, it can be speculated that the duration of the early stage of the phantom training may be shortened by additional training with a training tool such as the PhantHome. The tasks in the phantom course are learned from the easiest to the hardest; therefore, acceleration of the early stage of learning will accelerate the learning of the hardest tasks and attain earlier readiness for the final test. This could possibly improve final grades. Presumably, had the training tool been implemented throughout the whole course with additional complicated tasks (under development by our group), the transfer principle would influence all course exams. As such, a longer practice period with the tool could result in higher final grades, on average. From students’ attitude to the tool it was clear they trained on the tasks: they repeated the training 18.4±11.4 times. However, the time devoted by each student was not documented.

Fine motor skill is defined as the capacity to coordinate sensorial information and muscular response to perform a determined task [31]. For the small proportion of dentistry students that are clumsy with their hands, achieving a level of competence and safety is challenging. Considerable research in the field of dentistry investigated whether the manual performance of dental students is influenced more from innate ability or from the acquisition of skills that can significantly be improved by practice [32–35]. However, the results of these studies are conflicting, and no consensus can be reached. In the study of Schwibbe et al. [32] spatial and manual tests that were administered to dentistry candidates showed significant correlation with clinical performance. They concluded that both spatial and manual ability might predict performance in practical dentistry and should therefore be included in the application process for dental schools. On the other hand, Lundergan et al. [23] found that scores of manual tests were weak predictors of clinical performance compared with spatial ability test scores. Giuliani et al. [34] concluded that manual skill is not an aptitude, but can be improved substantially by practice. They showed that students who could follow training of the entire phantom course significantly improved in their manual ability. This finding is in agreement with research performed by Gansky [33] and Luck [2] who concluded that manual dexterity can be acquired and improved by means of exercise, and that ability tests should be mainly used for identifying the weakest students before pre-clinical courses, in order to offer them more training so that they may achieve the dental performances required.

During the last 20 years of the 20^th^ century, studies introduced various tools attempting to obtain additional acceptance screening to dental schools based on various exercises including tweezers [36–38]. However, no attempt to the best of our knowledge was done to develop a simple portable tool for home training that has influence on the clinical performances. Most of the academic studies in general require home exercises such as homeworks and preparation of seminars, for example. The present study shows that with the use of a non-expensive simple portable tool for home training, improvement of clinical perfomance can be achieved. This tool doesn’t require faculty supervision and does not require a turbine or other electrical tools that are used in the phantom laboratory. Moreover, with the increasing capabilities of 3D printing, more complicated tasks will probably develop for home training.

Nevertheless, despite the fact that Randomized Controlled Trial (RCT) is the best way to prevent selection bias and ensure that the experimental and control groups will be as similar as possible except for the intervention, our study was designed to use a historical control group, which is a subtype of RCT. The main reasons for this design are: 1. To prevent the possibility that students that may be assigned to the control group would try to obtain the interventional tool and practice at home, as they could have believed that this will improve their scores. 2. An ethical teaching issue: if one of the groups would have received lower scores, they could claim that the interventional tool was responsible for that. By choosing the historical control group, these issues were prevented. Future studies are required to further determine the effectiveness of the PhantHome tool in other schools.

## 5. Conclusions

1. Home training in the basic skills required in dentistry by the PhantHome tool parallel to the phantom course improves clinical performances in the early stages of phantom training.
2. Transfer principle, that is, training in one tool for improving capabilities in similar but not identical tasks exists in dental students training.

## Supporting information

S1 Appendix PhantHome training instruction. (DOCX)

S2 Appendix Characteristics of the experimental and control participants. (DOCX)
