# Supplementary material for: An interventional study for improving the manual dexterity of dentistry students
Source: PLoS One. 2019 Feb 1;14(2):e0211639. doi: 10.1371/journal.pone.0211639 (PMC6358065; doi:10.1371/journal.pone.0211639)
Supplement: S1 Appendix — (DOCX) [file pone.0211639.s001.docx]

Appendix A: PhantHome training instructions.

Students were instructed to insert the small pins using tweezer into the preparations using their dominant hand. They were advised to repeat the missions several times and to verify that the time required for the missions is reduced over training. They were instructed to start the training with direct vision and continue with the indirect vision using dental mirror. Then, they were advised to complicate the training by alternating tasks in each jaw and between the jaws, that is, using direct and indirect vision. (Table 1).

Table 1: PhantHome training instructions

|  | **Direct vision** |
| --- | --- |
| 1 | Tooth 11, labial |
| 2 | Tooth 46, mesio-buccal |
| 3 | Tooth 46, disto-buccal |
| 4 | Tooth 36, mesio-buccal |
| 5 | Tooth 36, disto-buccal |
| 6 | Tooth 46, mesio-lingual |
| 7 | Tooth 46, disto-lingual |
| 8 | Tooth 36, mesio-lingual |
| 9 | Tooth 36, disto-lingual |
|  |  |
|  | **Indirect vision** |
| 1 | Tooth 11, palatal |
| 2 | Tooth 16, occlusal |
| 3 | Tooth 16, mesio-buccal |
| 4 | Tooth 16, disto-buccal |
| 5 | Tooth 26, mesio-buccal |
| 6 | Tooth 26, disto-buccal |
| 7 | Tooth 16, mesio-palatal |
| 8 | Tooth 16, disto-palatal |
| 9 | Tooth 26, mesio-palatal |
| 10 | Tooth 26, disto-palatal |
