# Supplementary material for: An interventional study for improving the manual dexterity of dentistry students
Source: PLoS One. 2019 Feb 1;14(2):e0211639. doi: 10.1371/journal.pone.0211639 (PMC6358065; doi:10.1371/journal.pone.0211639)
Supplement: S2 Appendix — (DOCX) [file pone.0211639.s002.docx]

Appendix B

Table 2: Characteristics of the experimental (N=41) and control (N=59) groups at the beginning of the study.

| **Characteristic** | **Experimental group** | **Control group** | **P value^a^** |
| --- | --- | --- | --- |
| Age^b^ | 24.51 (2.2) | 25.46 (2.8) | 0.08 |
| Gender | F (n=26), M (n=15) | F (n=39), M (n=20) | 0.831 |
| Basic fine motor skills^c^ | 6.8 min (1.4) | 6.3 min (1.2) | 0.063 |
| Basic spatial perception^d^ | 23.8 min (8.7) | 23.2 min (7.8) | 0.687 |
| Cognitive ability^e^ | 732 score (32.2) | 725 score (18.9) | 0.264 |
| Hours of sleep at night^f^ | 6.6 h (0.8) | 6.3 h (1.01) | 0.102 |
| Dominant hand | 39 R, 2 L | 53 R, 6 L | 0.466 |

^a^Determined by use of the independent t test or the chi-square for group comparisons. ^b^Mean age and SD, ^C^Determined by the O'Connor direct test at T0, mean and SD,  ^d^Determined by the O'Connor indirect test at T0, mean and SD,
^e^Determined by the Psychometric score, mean and SD,
^f^Determined by the student questionnaire, mean and SD.
